# Neuroendocrine differentiation distinguishes basaloid variant of lung squamous cell carcinoma

**DOI:** 10.1186/s13000-022-01223-6

**Published:** 2022-05-10

**Authors:** Kianoosh Keyhanian, William J. Phillips, Benjamin S. Yeung, Marcio Gomes, Bryan Lo, Harmanjatinder S. Sekhon

**Affiliations:** 1 Department of Pathology and Laboratory Medicine, The Ottawa Hospital/Eastern Ontario Regional Laboratory Association, Critical Care Wing, Rm 4220, Box 117, 501 Smyth Road, Ottawa, ON K1H 8L6 Canada; 2 Department of Pathology and Laboratory Medicine, University of Ottawa, Faculty of Medicine, 451 Smyth Road, Ottawa, ON K1H 8M5 Canada; 3 Department of Medicine, Univeristy of Ottawa, Faculty of Medicine, Ottawa, ON K1H 8M5 Canada; 4 Department of Pathology and Laboratory Medicine, The Ottawa Hospital Research Institute, 501 Smyth Road, Ottawa, ON K1H 8L6 Canada

**Keywords:** Basaloid squamous cell carcinoma, Neuroendocrine differentiation, Gene expression, Immunohistochemistry, NOTCH

## Abstract

**Background:**

Neuroendocrine (NE) differentiation is widely studied in non-small cell lung carcinomas (NSCLC) however, its significance remains unclear in basaloid squamous cell carcinomas (B-SqCC). This study aims to assess the extent of NE differentiation in B-SqCC and characterize the underlying molecular process.

**Methods:**

This study evaluated resected B-SqCC, small cell lung cancer (SCLC) and poorly differentiated SqCC (PD-SqCC) from 2005 to 2020 at the Ottawa Hospital. Samples were subject to pathological review, immunohistochemistry (IHC) and survival analysis. Gene expression analysis was performed on B-SqCC samples exhibiting NE+ and NE- regions (paired samples) to identify differentially expressed genes (DEGs). These DEGs were subsequently validated in unpaired B-SqCC and TCGA samples.

**Results:**

B-SqCC cases were more likely to exhibit nuclear molding, resetting and peripheral palisading than PD-SqCC. B-SqCC were also more likely to demonstrate NE differentiation compared to PD-SqCC (*p* = 0.006). Pure basaloid squamous cell carcinoma (PB-SqCC) experienced poorer disease-free survival (HR = 3.12, *p* = 0.043) adjusted for stage.

Molecular characterization of paired B-SqCC samples demonstrated DEGs implicated in NOTCH signaling, SCLC and pulmonary neuroendocrine differentiation. Hierarchical clustering using discovered DEGs in unpaired B-SqCC samples distinguished tumors based on NE status (*p* = 0.048). Likewise, clustering The Cancer Genome Atlas (TCGA) samples with DEGs distinguished B-SqCC from SqCC samples (*p* = 0.0094).

**Conclusion:**

This study provides IHC and molecular evidence of significant NE-differentiation in B-SqCC and demonstrates their aggressive clinical behavior. These findings suggest that B-SqCC are biologically distinct from SqCC and share characteristics with SCLC.

**Supplementary Information:**

The online version contains supplementary material available at 10.1186/s13000-022-01223-6.

## Introduction

Basaloid squamous cell carcinoma (B-SqCC) is an aggressive histological subtype of lung cancer. In the most recent World Health Organization (WHO) classification of thoracic tumors, basaloid tumors of the lung were categorized as a variant of squamous cell carcinoma (SqCC), although previous WHO classifications considered them a separate entity [1]. Compared to other SqCC variants, B-SqCC are rare, making up 5–10% of all non-small cell lung cancer (NSCLC) cases [2, 3]. They are associated with poor prognosis, high rates of metastasis and poor overall survival compared to conventional SqCC [2, 4–6].

B-SqCC are not only challenging to treat, but difficult to recognize pathologically, as they share morphological features of poorly differentiated SqCC (PD-SqCC), small cell lung carcinoma (SCLC) and large cell neuroendocrine carcinoma (LCNEC) [7]. Their diagnosis is especially difficult on aspirate or small biopsy samples, where few cells are present. Immunohistochemistry (IHC) is a helpful diagnostic tool to differentiate these tumors, however, some overlap with other tumours poses challenges. The traditional IHC pattern of B-SqCC, like other SqCC, is p40 positive, TTF1 negative and neuroendocrine (NE) marker negative. However, variable immunohistochemical reactivity with TTF-1 and/or NE markers is observed clinically in a subset of B-SqCC cases.

NE differentiation occurs with low frequency in the context of NSCLC, described in up to 20% of cases without clear association with clinical outcome [8, 9]. To our knowledge, no study has characterized the extent of NE differentiation and studied its molecular drivers in B-SqCC. Further investigation is therefore warranted in B-SqCC given its distinctive histological features and aggressive behavior.

To fully describe experience at our center with B-SqCC and characterize the process of NE differentiation, this study was divided into 4 components. First, we reviewed histological and IHC features of B-SqCC, comparing to its morphologically resembling entities with basaloid cellular histology i.e. SCLC and poorly differentiated SqCC. Secondly, we performed a chart review of B-SqCC to evaluate clinical outcomes. Thirdly, we used gene expression data to determine genes differentially expressed in NE positive regions, compared to NE negative regions within single tumor samples. Gene expression differences were then validated in an independent cohort of B-SqCC and The Cancer Genome Atlas (TCGA) samples. Finally, we present an index case demonstrating a transition zone of B-SqCC to poorly differentiated neuroendocrine lung carcinoma that otherwise can be considered as a combined tumour.

## Methods

### Patients

Primary lung B-SqCC resected from 2011 to March 2020 were gathered from the Eastern Ontario Regional Laboratory (EORLA) pathology database at The Ottawa Hospital. All resected SCLC and a random subset of resected poorly differentiated SqCC (PD-SqCC) were selected as controls. Cases that received neoadjuvant chemoradiation were excluded.

### Clinical outcomes

Progression free survival was measured from the start of treatment until local disease recurrence, disease metastasis, or death. Patients who were discharged from follow-up were censored from subsequent analyses. Death was inferred based on documentation of death in the EMR or discontinuation of follow-up in patient with metastatic cancer without a previously specified reason. The relationship between variables and disease-free survival was assessed by the Proportional Cox Regression. Statistical significance was set at *p* < 0.05. Statistical analysis was performed on SPSS v25.0 for Mac.

### Histology and immunohistochemical review

Histological slides were reviewed by two thoracic pathologists and one senior pathology resident. Samples were included if consensus was achieved. A combination of histological and immunohistochemical features was used for the diagnosis of B-SqCC poorly differentiated SqCC (PD-SqCC). Cases with features overlapping with small cell/large cell neuroendocrine carcinoma or thoracic SMARCA4-deficient undifferentiated tumor were excluded (see more details in results section Histology and immunohistochemistry).

**Fig. 1:**
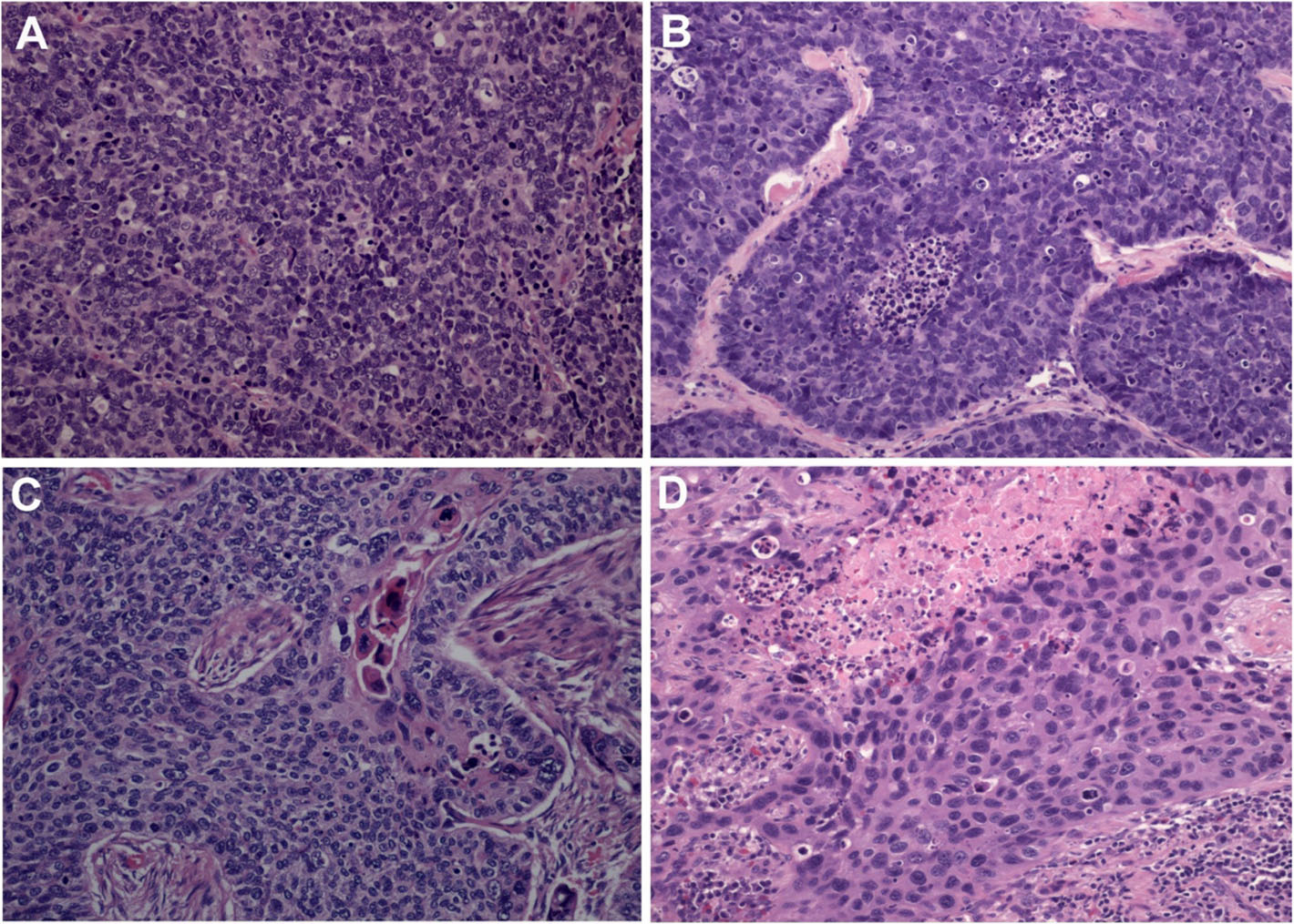
The photomicrograph shows morphological features of **A** small cell lung carcinoma, **B** pure-basaloid squamous cell carcinoma, **C** basaloid squamous cell carcinoma (focal <50% squamous cell differentiation) and **D** poorly differentiated squamous cell carcinoma

For the B-SqCC category, the percentage of squamous differentiation was assigned based on morphology. SqCC cases with more than 50% basaloid differentiation were classified as B-SqCC [1]. A subset of B-SqCC with no or less than 5% squamous differentiation were classified as pure basaloid squamous cell carcinoma (PB-SqCC) (Fig. 1a).

Histological characteristics were scored on ordinal scales. Nuclear pleomorphism was scored by the degree of atypia (1 = mild, 2 = moderate and 3 = severe), nucleoli by prominence (1 = inconspicuous, 2 = conspicuous and 3 = prominent) and necrosis by proportion of tumor (1 = 0–25%, 2 = 25–50%, 3 = 50–75% and 4 = > 75%).

The following IHC stains were employed: CK5, p40, TTF-1, chromogranin, synaptophysin and CD56. IHC stains were performed on the largest two tumor sections and cumulative ratios were recorded. Tumors with neuroendocrine marker positivity > 10% were considered neuroendocrine positive. A second NE marker was considered positive if it demonstrated > 5% tumor cell reactivity. The Fisher Exact test was employed to calculate the relationship between morphology and IHC characteristics and tumor type.

### Molecular analysis

Whole genome gene expression data was captured. A list of candidate genes was generated based on prior peer-reviewed evidence associating genes with pulmonary neuroendocrine differentiation.

Gene expression results were normalized to reads per million (RPM). Changes in expression were represented on a log_2_ scale. Genes were filtered out of the analysis if they did not meet a minimum expression level of 32 RPM in at least one B-SqCC sample. If more than one area was analyzed in a single region (in the case of technical replicates), the mean of expression values from that region were used.

B-SqCC samples were split into two groups for analysis: paired and unpaired. Paired samples contained both regions that expressed NE markers (NE+) and regions that did not (NE-). Paired sample analysis was appealing, as it allowed for comparison of intra-tumor gene expression profiles, thus controlling for the background gene expression differences. On the other hand, unpaired samples were either NE+ or NE-, but did not contain both regions. They represented inter-tumor comparisons between NE+ and NE- regions.

Differentially expressed genes (DEGs) were identified in paired B-SqCC samples by calculating the difference in gene expression between NE+ and NE- regions. Genes were considered differentially expressed if the absolute mean difference in gene expression was ≥1.5-fold or an absolute difference of ≥1.5 fold was identified in ≥3 samples.

Unpaired B-SqCC samples were used to validate DEGs and during hierarchical clustering. An absolute difference of ≥2.0-fold rather than ≥1.5-fold was set as the threshold for differential expression to adjust for the lower degree of background gene expression similarity. Hierarchical clustering was performed, using well described methodology [10]. Gene expression values were first log_2_ transformed as log_2_ (1 + RPM) and were centered around their means. Clustering was performed by Cluster 3.0 for Mac [11]. Fisher’s exact test was employed to determine whether statistical significance was achieved in clustering distributions. Statistical testing between cluster groups was performed by the Fisher’s Exact test.

### The cancer genome atlas data

Gene expression data from all 15 B-SqCC samples available at the time of collection and 30 randomly selected SqCC samples were downloaded from the TCGA portal (Firehose). The data was accessed in August 2018. Methodology for pathologic review along with DNA expression analysis for TCGA samples has previously been described [12].

## Results

### Patients

Fifty-four cases were included in the study. There were 26 B-SqCC (including 12 cases of PB-SqCC), 19 PD-SqCC and 9 SCLC cases. The mean age was 70 years (range = 53–79 years) in patients with B-SqCC, 69 years (range = 55–79 years) in PD-SqCC and 71 years (range = 54–88 years) in SCLC. Likewise, the gender distribution was 35% (*n* = 9), 42% (*n* = 8) and 56% female (*n* = 5), respectively.

### Clinical outcome

Overall, mean follow-up was 22 months (range = 1–120 months). In patients with B-SqCC, there were 3 (12%) mortalities, 11 (42%) patients with disease recurrence or metastasis, and 12 (46%) with no event. Likewise, in patients with PD-SqCC, there was 1 (11%) mortality, 5 (26%) patients with disease recurrence or metastasis, and 13 (68%) with no event. Patients with PB-SqCC had the highest event rate. There was 1 (8%) mortality, 7 (58%) patients with recurrence or metastasis, and only 4 (33%) with no event. Controlling for stage, patients with B-SqCC demonstrated a trend towards poorer progression-free survival (HR = 2.16, 95%CI = 0.82–5.48, *p* = 0.13) compared to patients with PD-SqCC. Patients with PB-SqCC experienced significantly worse progression-free survival (HR = 3.12, 95%CI = 1.03–9.39, *p* = 0.043) adjusted for stage, compared to PD-SqCC (Suppl Fig. 1).

### Histology and immunohistochemistry

Morphological features were assessed in each tumor type (Fig. 1). Nuclear molding and peripheral palisading occurred more frequently in B-SqCC compared to PD-SqCC (*p* = 0.021; *p* < 0.001 respectively) while nuclear pleomorphism and nucleoli were less prominent in B-SqCC than in PD-SqCC (*p* = 0.003; *p* = 0.001 respectively) (Table 1).

**Table 1:** Histological characteristics by tumor type

Histology	Zonal Necrosis *(scale 1–3)*	Nuclear Molding *(%)*	Rosettes *(%)*	Peripheral Palisading *(%)*	Nucleoli *(scale 1–3)*	Nuclear Pleomorphism *(scale 1–3)*
PB-SqCC (*n* = 12)	2.2	50%	50%	83%	1.7	2.1
All B-SqCC (*n* = 26)	2	46%	35%	92%	1.6	2.2
PD-SqCC (*n* = 19)	1.9	10%	0	37%	2.3	2.7
SCLC (*n* = 9)	1.4	88%	67%	78%	1.4	1.7

PB-SqCC (Pure basaloid squamous cell carcinoma), B-SqCC (Basaloid squamous cell carcinoma), PD-SqCC (Poorly differentiated squamous cell carcinoma), SCLC (Small cell carcinoma)

The pattern of neuroendocrine expression by IHC ranged from small discrete areas to full block positivity and stain intensity ranged from very weak to high intensity (Table 2). Overall, 65% (17/26) of B-SqCC and 67% (8/12) of PB-SqCC demonstrated > 10% NE reactivity with one NE marker compared to 21% (4/19 cases) of PD-SqCC (*p* = 0.006, *p* = 0.031, respectively). Positivity for more than one NE marker was seen in 27% (7/26) of B-SqCC cases, while no PD-SqCC cases were positive for more than one NE marker.

**Table 2:** IHC marker reactivity by tumor type

IHC Reactivity	Chromogranin (> 10%)	Synaptophysin (> 10%)	CD56 (> 10%)	Chromogranin (> 70%)	Synaptophysin (> 70%)	CD56 (> 70%)
*PB-SqCC (*n* = 12)	17%	8%	50%	0%	0%	17%
B-SqCC (*n* = 26)	19%	12%	56%	0%	0%	15%
PD-SqCC (*n* = 19)	0%	0%	21%	0%	0%	0%
SCLC (*n* = 9)	88%	100%	100%	88%	100%	100%

PB-SqCC (Pure basaloid squamous cell carcinoma), B-SqCC (Basaloid squamous cell carcinoma), PD-SqCC (Poorly differentiated squamous cell carcinoma), SCLC (Small cell carcinoma)

We also compared reactivity with other IHC markers routinely used in the diagnostic workup of SqCC. Interestingly, B-SqCC demonstrate lower expression of p40 with 19% of cases showing < 10% positivity, while 23% of B-SqCC cases showed > 10% positivity with TTF1 (Suppl Table 1) As stated in Methods Section Histology and immunohistochemical review, cases with features overlapping with small cell/large cell neuroendocrine carcinoma or thoracic SMARCA4-deficient undifferentiated tumor were excluded. Therefore, cases with TTF1 positivity were enrolled as B-SqCC if they had also high (> 70%) expression of p40 and CK5, while cases with low p40, were enrolled as B-SqCC if negative for TTF1, showed negative or low expression of NE markers (< 10%) and showed CK5 positivity (20 to 100% in our cohort).

### Identification of differentially expressed genes (DEGs)

One hundred sixty four candidate genes, the expression of which is involved in neuroendocrine phenotype, were identified based on our literature review [12–15]. Of these, only 102 (62.2%) met the minimum read count. There were 6 paired samples, which contained both NE+ and NE- areas within the same tumor. They were composed of 4 (67%) PB-SqCC, 1 (16.6%) B-SqCC and 1 (16.6%) mixed B-SqCC/SCLC (index case).

**Fig. 2:**
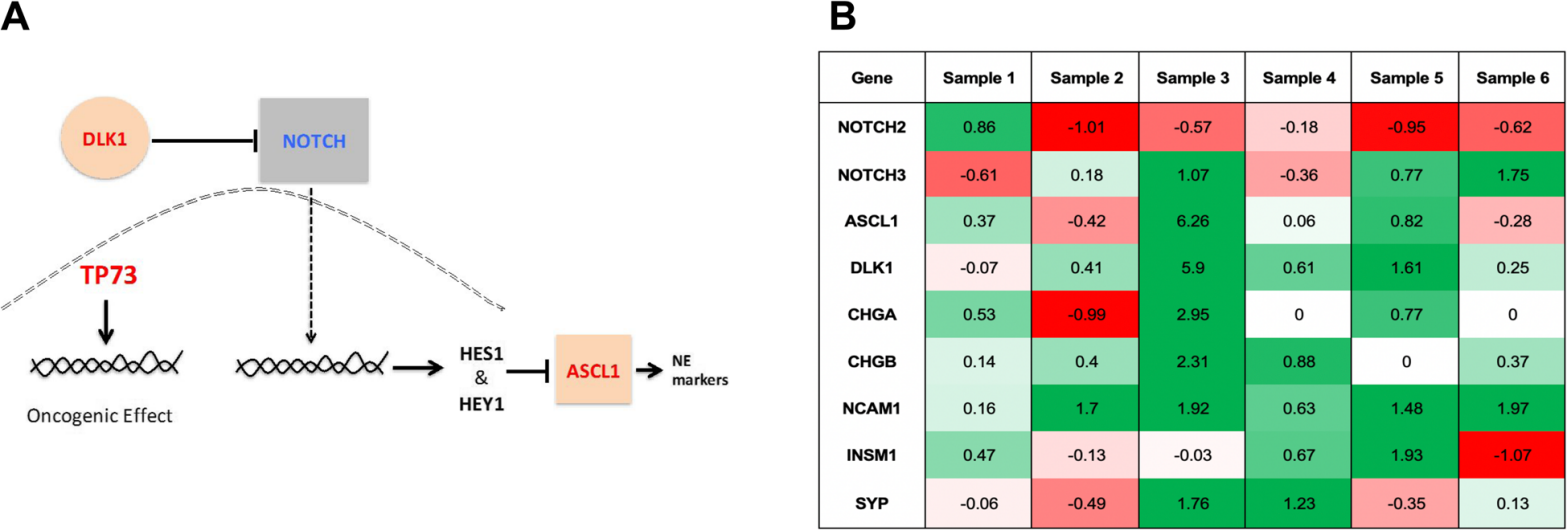
**A** A Overview of NOTCH and TP73 signaling. Arrows indicate activation, while blunt ends indicate inhibition. Note, upregulation of DLK1 inhibits NOTCH activity, while ASCL1 is inhibited by NOTCH. TP73 is upregulated in areas of neuroendocrine positivity and contributes to oncogenesis through an alternative pathway. **B** Heat map depicting differences in gene expression (log_2_ fold-change) of genes in the NOTCH pathway in 6 paired B-SqCC samples. Red signifies lower expression, while green signifies heightened expression in neuroendocrine regions. In 5/6 samples, upstream inactivation (low expression of NOTCH2 or high expression of ASCL1, DLK1 or NOTCH3) with corresponding upregulation downstream neuroendocrine markers (CHGA, CHGB, NCAM1, INSM1 and SYP) is observed of genes in the NOTCH pathway. Red signifies lower expression, while green signifies heightened expression in neuroendocrine regions. In 5/6 samples, upstream inactivation (low expression of NOTCH2 or high expression of ASCL1, DLK1 or NOTCH3) with corresponding upregulation downstream neuroendocrine markers (CHGA, CHGB, NCAM1, INSM1 and SYP) is observed

Thirty genes were differentially expressed in paired samples (Table 3). Notably, downregulation of EP300, RBL2, PTEN and CREBBP and upregulation of TP73 and EEF1A2 was identified. These genes are known to play a role in the pathogenesis of SCLC ^20^. Downregulation of NOTCH2 and upregulation of DLK1, ASCL1, NOTCH3 and CHGB was also observed and are signs of NOTCH pathway inhibition (Fig. 2a).

**Table 3:** Genes associated with neuroendocrine differentiation that showed differential expression in neuroendocrine positive areas in basaloid squamous cell carcinoma samples

Gene	Change in expression (mean log_2_ fold-change)	# of samples with differential expression (*n* = 6)
**Under-expressed genes**		
*NOTCH pathway genes*		
NOTCH2	−0.413300811	3
*SCLC associated genes*		
ADNP	−1.092576002	3
EP300	−1.010211564	3
KIF1A	−0.894186601	3
RBL2	−0.767802098	2
PTEN	−0.733479705	3
MED13L	−0.643618453	2
MDM2	−0.629119971	2
CREBBP	−0.291361084	3
*Other neuroendocrine genes*		
INA	−0.742248345	3
MAD2L1	−0.702589626	2
IGF1R	−0.349525122	3
**Over-expressed genes**		
*NOTCH pathway genes*		
DLK1	1.450305483	3
NCAM1	1.309526048	5
ASCL1	1.136928289	2
CHGB	0.682839331	2
NOTCH3	0.465878996	3
HEY1	0.298499747	3
*SCLC associated genes*		
TP73	1.642280684	5
MCM4	1.127153984	4
CADM1	0.66578049	2
EEF1A2	0.55101448	5
*Other neuroendocrine genes*		
SST	1.407048734	2
BARX1	1.209322164	4
CEACAM5	1.1847035	3
SCGB3A1	0.966463878	3
CALML3	0.728573672	4
TMSB15A	0.532894103	3
PRAME	0.596517667	2
GNG4	0.485250927	3

Evidence of NOTCH pathway inhibition was identified in 83.3% (5/6) of paired B-SqCC samples, as evidenced by either upregulation of ASCL1 or DLK1, or downregulation of NOTCH2 or HEY2. In all samples with evidence of NOTCH pathway inhibition, at least 1 downstream marker of NE differentiation (NCAM1, CHGA, CHGB, SYP, INSM1) was upregulated. In 60% of samples (3/5), 3 or more downstream markers of NE differentiation were upregulated (Fig. 2b).

### Validation of differentially expressed genes (DEGs)

There were 9 unpaired B-SqCC samples, entirely composed of NE+ or NE- cells, included in the study. Of those, 4 (44%) were NE+ and 5 (56%) were NE-. Gene expression analysis of unpaired B-SqCC samples reproduced many of the findings identified in the paired sample analysis, such as decreased gene expression in KIF1A and increased gene expression in CEACAM5, EEF1A2, CHGB, CALML3 and TP73 in NE+ samples (Supple Table 2).

**Fig. 3:**
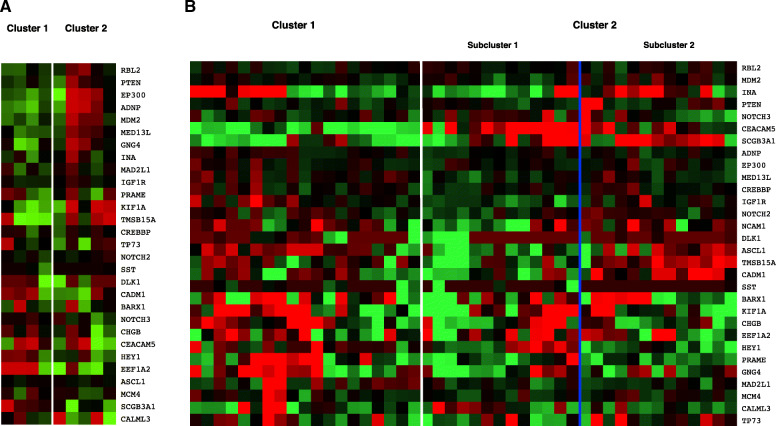
Hierarchical clustering using differentially expressed genes. Red indicates decreased expression, while green indicates heightened expression. **A** Hierarchical clustering of unpaired basaloid squamous cell carcinoma samples distinguishes tumors based on immunohistochemical NE status. Cluster 1 is enriched for NE negative (by IHC) samples (0/4), while Cluster 2 is enriched in NE positive tumors (4/5) (*p* = 0.048). **B** Hierarchical clustering of TCGA samples distinguished cases based on tumor type. Cluster 1 is enriched with non-basaloid squamous cell carcinomas (2/19 are basaloid type), while Cluster 2 is enriched with basaloid squamous cell carcinomas (13/26 are basaloid type) (*p* = 0.0094). A subset of cases from Cluster 2 (Sub-cluster 1) demonstrate higher expression of NOTCH pathway genes including DLK1 (log_2_ FC = 2.8), ASCL1 (log_2_ FC = 1.8), CHGB (log_2_ FC = 1.0) and NCAM1 (log_2_ FC = 2.9)

Hierarchical clustering, using the 30 DEGs identified in the paired analysis, was performed on the unpaired B-SqCC samples. Hierarchical clustering distinguished cases by their corresponding immunohistochemical NE status (*p* = 0.048) (Fig. 3a). This result provides further molecular support for NE differentiation in NE+ B-SqCC cases suggested by IHC. The gene expression profile of NE+ cases demonstrated upregulation of NOTCH3, CHGB, CEACAM5 and EEF1A2 as well as downregulation of RBL2, PTEN, EP300, ADNP and KIF1A. NCAM1 was removed from the analysis, as it was used as an IHC marker to determine the NE status of the cases.

### Hierarchical clustering of TCGA samples

Hierarchical clustering, using the same DEGs as above, was performed on TCGA samples. In total, 15 B-SqCC and 30 random SqCC TCGA samples were included. Hierarchical clustering of TCGA samples using the NE-implicated DEGs distinguished cases by tumor type (B-SqCC versus SqCC) (*p* = 0.0094) (Fig. 3b). Interestingly, higher expression of ASCL1, DLK1, CHGB and NCAM1 was observed in a subset of cases in the B-SqCC enriched cluster. Heightened expression of these 4 genes is associated with NOTCH pathway inhibition.

### The index case: a diagnostic dilemma of a single tumor with both B-SqCC and SCLC features

**Fig. 4:**
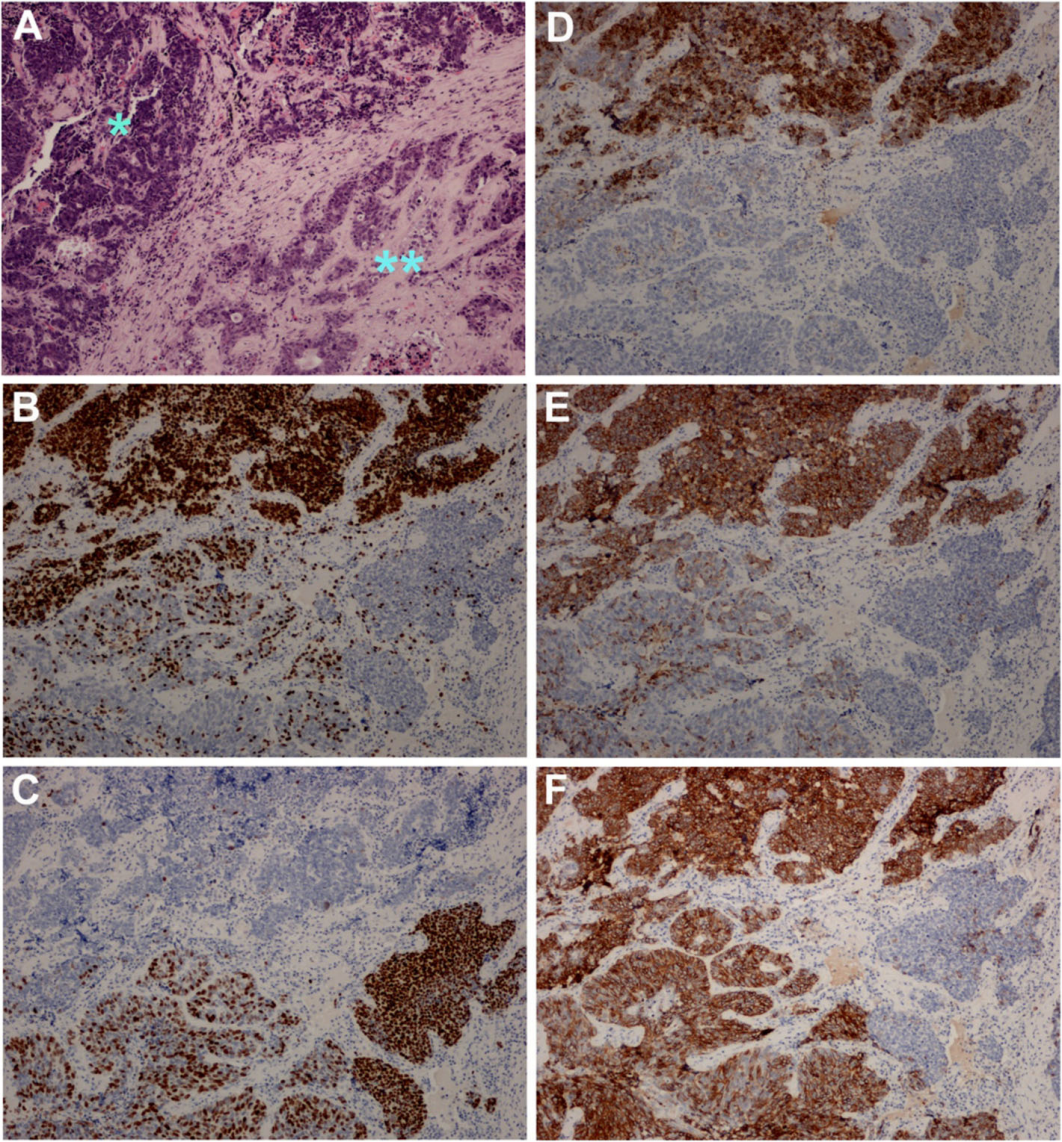
The photomicrographs show the morphological features and immunohistochemical profile of a tumor exhibiting small cell lung carcinoma (SCLC) morphology transitioning to basaloid carcinoma (B-SqCC) features. **A** The SCLC characterized by hyperchromatic densely packed cells with scant cytoplasm on the left (*) and pure B-SqCC with hyperchromatic nuclei surrounded by moderate eosinophilic cytoplasm on the right (**). The immunohistochemical stains highlight the transitioning spatial differentiation with **B** TTF-1 positive staining in SCLC and negative staining in pure B-SqCC and conversely **C** p40 negative staining in SCLC and strong positive in pure B-SqCC. On the other hand neuroendocrine stains including **D** Chromogranin, **E** Synaptophysin and **F** CD56 are positive in SCLC while pure B-SqCC is either focally positive or is negative

The index case is a 2.8 cm, pT2N2 tumor separated into three regions based on morphological and immunohistochemical characteristics: (1) PB-SqCC without NE differentiation, (2) PB-SqCC with NE differentiation and (3) SCLC (Fig. 4). On histology, the tumor consists of nests and clusters of basaloid-looking cells with intermediate-sized pleomorphic nuclei, a small rim of cytoplasm and shows peripheral palisading, molding, and focal rosetting. Zonal necrosis was not present, however, there was abundant individual cell apoptosis. Parts of the tumor demonstrated p40 and CK5 positivity, together with negative to patchy neuroendocrine positivity (60% CD56, 30% chromogranin and 50% reactivity with synaptophysin). These areas merged in a continuous fashion with another part of the tumor showing no p40/CK5 positivity as well as diffuse and strong CD56, Chromogranin and synaptophysin reactivity. Correlating with IHC, the histological features were quite similar in the basaloid-like area compared to the SCLC-like area except for the fact that SCLC clusters were very compact with marked nuclear molding and no discernable cytoplasm.

Gene expression analysis was performed on each of the three regions. SCLC and NOTCH pathway genes such as ASCL1, DLK1, CEACAM5, SST, CHGB, NCAM1, TP73, EEF1A2, TMSB15A, INA and HEY1 showed lowest expression in the B-SqCC NE- region, intermediate expression in the PB-SqCC NE+ region and highest expression in the SCLC region. PTEN and NOTCH2 showed the opposite pattern with highest expression in the PB-SqCC NE- region and lowest expression in the SCLC region.

## Discussion

In our cohort of 26 B-SqCC, including 12 cases of PB-SqCC, we observe high rates of NE differentiation. By IHC, 65% of B-SqCC samples showed positivity with at least one NE marker, while 27% of cases were positive with more than one marker. Gene expression results complimented IHC findings revealing upregulation of NCAM1, CHGB and to a lesser degree, CHGA. Interestingly, hierarchical clustering of TCGA samples using genes associated with NE differentiation, separates B-SqCC from other SqCC, suggesting differential expression of NE genes in B-SqCC. We also show that PB-SqCC are associated with worse progression free survival than PD-SqCC adjusted for stage at diagnosis, which is in keeping with its aggressive clinical behavior. Given that NE differentiation is poorly understood in basaloid tumors and appears to be frequently identified, we believe this study contributes meaningfully to the current understanding of the pathological and molecular biology of B-SqCC.

### Neuroendocrine differentiation in basaloid squamous cell carcinomas

In clinical practice, there is commonly focal positivity for NE markers in basaloid neoplasms. Although this phenomenon is not well described, it has been reported in the literature [16]. To date, its biological mechanism has not been elucidated. In our cohort, 65% of B-SqCC show evidence of NE differentiation by IHC, most commonly by positivity in CD56. That said, in about 50% of the cases positive for CD56, another NE marker was also expressed in ≥5% of neoplastic cells. Although not entirely specific, strong and diffuse CD56 positivity is a sensitive marker for SCLC and other neuroendocrine neoplasms of the lung [17, 18]. We acknowledge that CD56 reactivity may not indicate NE differentiation specifically, however, our data supports NE associated gene expression alterations such as NOTCH pathway inhibition in areas highlighted by NE IHC markers. Hierarchical clustering of unpaired B-SqCC samples using NE associated gene expression correlated well with IHC NE positivity, providing support for NE differentiation at the gene expression level (Fig. 3a).

The NOTCH pathway appears to contribute to NE differentiation in B-SqCC in an important way. In general, downregulation of NOTCH signaling is associated with NE differentiation in pulmonary neoplasms; recent data suggests that 25% of SCLC have alterations to the NOTCH pathway [12, 13, 19, 20]. The mechanism of NOTCH signaling has been previously described and is outlined in Fig. 2a. Briefly, the NOTCH pathway contains four distinct NOTCH genes: NOTCH1–4, each encoding a NOTCH transmembrane receptor. DLK1 has been shown to be a direct inhibitor of NOTCH genes in vitro, with emerging evidence of upregulation in vivo in pulmonary neuroendocrine neoplasms [21]. ASCL1 is inhibited by activation of the NOTCH pathway and induces NE differentiation when expressed at higher levels [14]. HEY1, HEY2, HES1 and HES2 are key mediators in the NOTCH pathway [21].

Inhibition of the NOTCH pathway was found in most of our B-SqCC samples, mainly observed through downregulation of NOTCH2 or upregulation of ASCL1 and DLK1. Differential expression was not observed in HES1 or HES2, although HEY2 was mildly under-expressed and HEY1 was over-expressed. This suggest that other mediators may be contributing to NOTCH signaling in B-SqCC. Another intriguing finding was upregulation of NOTCH3. NOTCH3 is unique, as it has been shown to be upregulated in non-small cell lung carcinoma in vivo [22]. In vitro, there is evidence that down-regulation of NOTCH3 is associated with reduced cell proliferation and cell motility in human lung cancer cell lines [23]. Thus, NOTCH3 may have a different role in NE differentiation than NOTCH1 and NOTCH2, which are downregulated in NE+ tumors.

Other genes associated with NE differentiation in our sample were CEACAM5, SST, TMS15A, GNG4, INA and MAD2L1. Each of these genes has been linked with NE differentiation in lung cancers previously [12–15]. In particular, CEACAM5 is part of the carcinoembryonic antigen family and upregulation has previously been linked with SCLC and large cell neuroendocrine carcinoma by IHC [24].

### Basaloid SqCC shares morphologic and molecular features with small cell lung carcinoma

B-SqCC are morphologically similar to SCLC and share their aggressive clinical behavior. Our findings indicate that the gene expression profile driving NE differentiation in basaloid tumors mimics key molecular changes found in SCLC. Specifically, we report upregulation of EEF1A2 and downregulation of EP300, RBL2, CREBBP and PTEN in B-SqCC samples showing NE positivity. In SCLC, EP300 and CREBBP harbor high rates of mutations and inactivating translocations, whereas under-expression of RBL2 was associated with decrease latency to development of SCLC in murine models [12]. PTEN is potent tumor suppressor, whose loss of expression or impaired function is strongly implicated in the pathogenesis of SCLC [25]. TP73, specifically, shows a strong signal of over-expression in NE+ B-SqCC. TP73 plays an important role in the development of malignant glioma and hepatocellular carcinoma [26, 27]. In lung cancers, increased expression of TP73, has been observed independent of expression changes to TP53 [28]. More recently TCGA published a study demonstrating the significance of genetic rearrangements in TP73 to the molecular landscape of SCLC [12].

In this study, we focused on the spectrum of tumors that are characterized by basaloid cellular morphology with scant cytoplasm and high nuclear to cytoplasmic ratio i.e. B-SqCC, PD-SqCC and SCLC. However, in clinical practice, large cell neuroendocrine carcinoma (LCNEC) also frequently enters the differential diagnosis. LCNEC can be encountered alone or in combination with NSCLC or SCLC. It’s pathogenesis also has considerable overlap with SCLC, including a subgroup with SCLC-like molecular profile (RB and P53 inactivation, MYCL amplification) [1]. Diffuse expression of squamous markers and focal to no NE reactivity distinguishes B-SqCC from LCNEC. However, the possibility of combined B-SqCC with LCNEC should be considered in B-SqCC with more significant NE positivity. LCNEC was ruled out in B-SqCC cohort in which 85 and 77% of cases showed diffuse (> 70%) CK5/6 and p40 positivity, respectively.

When encountering a poorly differentiated lung neoplasm, another diagnostic consideration would be “thoracic SMARCA4-deficient undifferentiated tumor”. The histological morphology in these cases is characterized by sheets of poorly cohesive round to ovoid cells with eccentric nuclei and prominent nucleoli. About 85% of our B-SqCC cases had diffuse (> 70%) CK5/6 positivity which would be unusual in SMARCA4-deificient tumor. Correlating morphology, IHC and molecular data in B-SqCC cases, our results did not support a diagnosis of “thoracic SMARCA4-deficient undifferentiated tumor” in any of these cases.

The index case is a unique example of the histologic resemblance of PB-SqCC and SCLC. This sample lacks significant expression of squamous cell markers except for p40 positivity in PB-SqCC-like zone. In addition, it shows a gradient of increasing NE expression in transition zones between B-SqCC and SCLC. This gradient correlates with IHC expression patterns, and gene expression data, which suggests a phenotypical transformation as B-SqCC approaches a “SCLC phenotype”. These findings challenge the elimination of PB-SqCC as a separate entity from current nomenclature, considering that PB-SqCC may be more akin to poorly differentiated neuroendocrine carcinoma rather than poorly differentiated squamous cell carcinoma. We anticipate further understanding of NE differentiation in B-SqCC will help in accurate histological identification and may lead to tumor-specific therapy.

### Comparing and distinguishing basaloid squamous cell carcinomas with poorly differentiated squamous cell carcinomas

PD-SqCC was selected as a control group for comparison of histological morphology, rate of NE differentiation and prognosis with B-SqCC. There is significant overlap in the morphological features between entities with basaloid cellular morphology. For diagnosis of PD-SqCC, a combination of morphological and immunohistochemical features were used (Tables 1 and 2, Suppl Table 1). We considered “large-cell neuroendocrine carcinoma” in the differential diagnosis of our PD-SqCC cases. CD56 was focal/patchy positive in 4 out of 19 cases in PD-SqCC (about 10% positivity in each case) and no case showed a second NE marker reactivity.

The results from the hierarchical clustering of TCGA cases are remarkable. They distinguish B-SqCC from a cohort of randomly derived SqCC using genes associated with NE differentiation. This aligns with our IHC findings showing higher levels of NE positivity in basaloid samples and supports the hypothesis that NE differentiation might be an important distinction between B-SqCC and SqCC.

Interestingly, other groups have investigated the molecular profile of basaloid lung neoplasms. *Li* et al. was one of the first groups to do so in 2004 [16]. Their group performed a supervised hierarchical clustering analysis of gene expression data from a heterogenous sample of NSCLC including large cell neuroendocrine carcinomas, adenocarcinomas, SqCC and B-SqCC. Using the expression data from 25 genes, they showed that B-SqCC samples formed a discrete cluster, while the other NSCLC subtypes were not uniquely classifiable. Later, *Brambilla* et al. subclassified SqCC samples by mRNA expression profiles and concluded that B-SqCC represent a distinct histomolecular entity from SqCC [6]. These histologic and molecular discrepancies, including differences in NE differentiation, are significant in current clinical practice considering B-SqCC and non-basaloid SqCC are largely treated as single entity.

### Limitations

This is a single-centered, retrospective study. We acknowledge that we present data on a small number of B-SqCC. This is attributed to the rarity of B-SqCC, specifically PB-SqCC, in the general population. To improve the quality of our analysis on a small sample size, we used paired B-SqCC analysis to control for background gene expression differences and optimize gene expression signals associated with NE differentiation. Analysis of TCGA samples was restricted by the availability of metadata, for example we were unable to determine whether samples demonstrated NE differentiation by immunohistochemistry. Finally, in any large throughput gene expression analysis study, multiple hypothesis testing must be considered. We balanced the competing interests of unbiased analysis and multiple hypothesis testing by focusing the analysis on a list of candidate genes of interest.

## Conclusion

Basaloid SqCC frequently exhibit NE differentiation. Our results indicate that genes involved in the NOTCH pathway and in SCLC play an important role in NE differentiation within B-SqCC. These findings suggest that NE differentiation may have more important implications in B-SqCC compared to other NSCLC. Our data support the hypothesis that B-SqCC are distinct from simply poorly differentiated SqCC; while sharing immunohistochemical and molecular characteristics with SCLC. This study sets up a stage for future work aimed at further understanding the role of NE differentiation in B-SqCC that may help promote more accurate molecular-pathological diagnosis and develop basaloid-specific treatment strategies.

## Supplementary Information

**Additional file 1.**

## Data Availability

The datasets used and/or analysed during the current study are available from the corresponding author on reasonable request.

## Abbreviations

NE: Neuroendocrine
NE +: Neuroendocrine positive.
NE-: Neuroendocrine negative
B-SqCCa: Basaloid squamous cell carcinoma
PB-SqCCa: Pure basaloid squamous cell carcinoma
PD-SqCCa: Poorly differentiated squamous cell carcinoma
SCLC: Small cell lung cancer
