# Supplementary material for: Neuroendocrine differentiation distinguishes basaloid variant of lung squamous cell carcinoma
Source: Diagn Pathol. 2022 May 10;17:46. doi: 10.1186/s13000-022-01223-6 (PMC9088121; doi:10.1186/s13000-022-01223-6)
Supplement: Supplementary file 1 — Additional file 1. [file 13000_2022_1223_MOESM1_ESM.docx]

**SUPPLEMENTARY METHODS**

**Supplementary Method 1: RNA extraction and Genomic Profiling**

In B-SqCC cases, dissectible areas of neuroendocrine differentiation were marked on the slide to be separated from NE negative areas for RNA extraction. RNA was isolated from the sections of formalin-fixed paraffin embedded (FFPE) tumour blocks from tumour resection specimens. Unstained 10μm - thick sections on slides were prepared from FFPE blocks. Using pathologist-annotated H&E slides as a reference, tumour-containing regions (tumour cellularity >80%) from the unstained sections were then micro-dissected into mineral oil with the Avenio Millisect (an automated tissue dissection instrument). Samples were transferred to a 1.5mL microcentrifuge tube, deparaffinized in 300μl of mineral oil and heated to 80°C for 30 seconds. Genomic RNA was isolated using the RecoverAll Total Nucleic Acid Isolation kit (Invitrogen) with an in-house modified protocol. The tissue was digested using 104μl of Digestion Reaction mix (4μl Protease mix, 25μl digestion buffer and 75μl nuclease-free water), followed by one-hour incubations at 55°C and 90°C. The aqueous phase was carefully transferred to a PureLink filter column and isolation was continued as per the manufacturer’s protocol. RNA concentration was quantified via Qubit 3.0 (Life Technologies).

**SUPPLEMENTAL FIGURES**


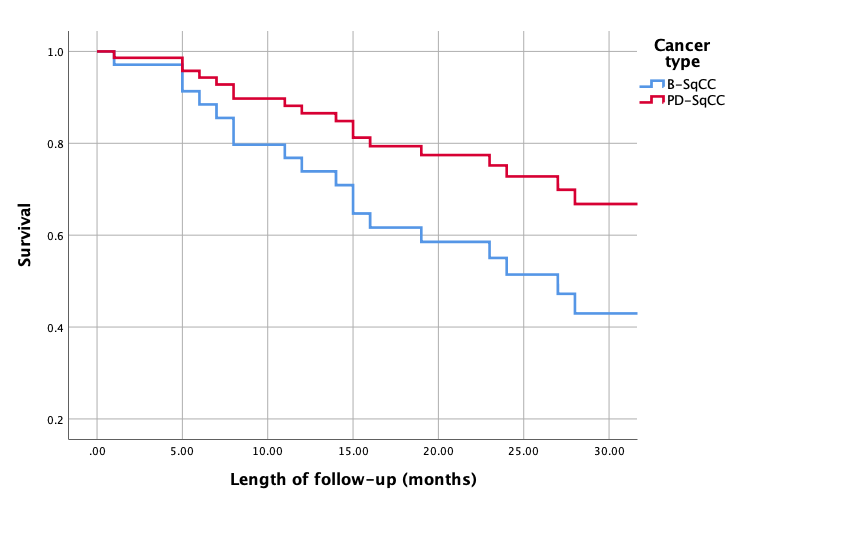


**Supplemental Figure 1:** Proportional Cox Regression comparing the proportion of patients without an adverse event between PB-SqCC and PD-SqCC. Analysis is adjusted for stage at presentation. Hazards ratio for adverse event in B-SqCC is 3.12 (p<0.05).

**SUPPLEMENTAL TABLES**

**Supplemental Table 1:** SqCC markers and TTF1 immunohistochemistry in the study groups*.

| SqCC phenotype SCLC phenotype | | | | | | | |
| --- | --- | --- | --- | --- | --- | --- | --- |
|  | **CK5 (>90%)** | **p40 (>90%)** | **TTF1 (<10%)** | | **CK5 (<10%)** | **p40 (<10%)** | **TTF1 (>90%)** |
| Pure B-SqCC (n = 12) | 75% | 58% | 92% |  | 0% | 17% | 0% |
| All B-SqCC  (n = 26) | 77% | 58% | 77% |  | 0% | 19% | 4% |
| PD-SqCC (n = 19) | 100% | 79% | 79% |  | 0% | 0% | 0% |
| SCLC (n = 9) | 0% | 0% | 0% |  | 56% | 100% | 100% |

* The results are categorized based on similarity to the two major categories in differential diagnosis of B-SqCC, i.e. SqCC with high expression of CK5 and p40 and low TTF1 compared to SCLC with low expression of CK5 and p40 and high TTF1.
